# Indications for home mechanical ventilation in children: changes over four decades

**DOI:** 10.1007/s00431-025-06559-x

**Published:** 2025-10-28

**Authors:** Laura Verweij van den Oudenrijn, Michael Gaytant, Lieke Arts, Peter Wijkstra, W. Ludo van der Pol, Esther Veldhoen

**Affiliations:** 1 Pediatric Intensive Care Unit and Center of Home Mechanical Ventilation, Wilhelmina Children’s Hospital, University Medical Center Utrecht, 3508 AB, Utrecht, 85090 The Netherlands; 2 Center of Home Mechanical Ventilation, University Medical Center Utrecht, Utrecht, the Netherlands; 3 Department of Pulmonary Diseases/Home Mechanical Ventilation, University of Groningen, University Medical Center Groningen, Groningen, the Netherlands; 4 University of Groningen, University Medical Center Groningen, Groningen Research Institute for Asthma and COPD, Groningen, The Netherlands; 5 Department of Neurology, Brain Center Rudolf Magnus, University Medical Center Utrecht, Utrecht, The Netherlands

**Keywords:** Pediatrics, Cohort studies, Respiration, artificial, Home care services, Hospital-based

## Abstract

Home mechanical ventilation (HMV) has been used for more than 40 years in the Netherlands. With this retrospective single-center study, we aimed to evaluate the demographics of children supported by HMV and changes in indications and type of support. We retrieved relevant data of all patients younger than 19 years who started HMV between 1979 and 2023 from a prospective database and from medical notes. A total of 544 children started with HMV (median age 13.1 years (IQR 5–16)). After a rapid increase between 1979 and 2017, the number of children treated with HMV stabilized. Nearly 40% of patients started HMV unplanned, because of failed weaning off the ventilator after surgery or an infection. Thirty percent of all children started with invasive ventilation. A majority of patients had a neuromuscular disorder (NMD) (*n* = 305, 56%) or a genetic syndrome (*n =* 85, 16%). The relative contribution of other diseases than NMDs increased in the 21th century. The relative number of patients younger than 5 years using HMV increased significantly over the years, from 13% in 1989–1998 to 30% in 2014–2022. This increase was even more pronounced for children using non-invasive ventilation (NIV), i.e. < 4% till 2003 and 30% in 2019–2022. *Conclusion*: This large longitudinal cohort study shows that the number of children requiring HMV remains stable but that the relative number of young children using HMV and the causes of chronic respiratory failure changed over the years. Also, NIV became the predominant mode in children starting HMV.

**Table:** 

**What is Known:** • *The number of children with home mechanical ventilation increased over the last decades.* • *Neuromuscular diseases are the most common reason for starting home mechanical ventilation.*
**What is New:** • *The number of children requiring home mechanical ventilation remained stable in recent years.* • *The relative number of children younger than 5 years using home mechanical ventilation, in particular non-invasive ventilation, increased over the years.* • *Causes of chronic respiratory failure necessitating home mechanical ventilation changed over the years.*

**What is Known:**

• *The number of children with home mechanical ventilation increased over the last decades.*

• *Neuromuscular diseases are the most common reason for starting home mechanical ventilation.*

**What is New:**

• *The number of children requiring home mechanical ventilation remained stable in recent years.*

• *The relative number of children younger than 5 years using home mechanical ventilation, in particular non-invasive ventilation, increased over the years.*

• *Causes of chronic respiratory failure necessitating home mechanical ventilation changed over the years.*

## Introduction

Home mechanical ventilation (HMV) is used for patients with a range of disorders that cause respiratory failure. Long term mechanical ventilation was first introduced in the Netherlands in 1956 during a poliomyelitis epidemic [1]. Some patients remained ventilator-dependent, which required adjustments that would eventually allow patients to be discharged. In 1960, the first adult patient was discharged with HMV [2].

HMV started as negative pressure ventilation, until technological advances allowed invasive positive pressure ventilation (IPPV) via tracheostomy in the 1980 s and non-invasive ventilation (NIV) since 1990. NIV eventually became the most common approach.

In 1979, the first child was discharged with HMV after being admitted for 400 days with a diagnosis of traumatic spinal cord injury (SCI). Nowadays, HMV is used in children with a heterogeneous group of disorders. In the Netherlands, care is centralized in 4 centers of HMV [3, 4].

Although the history of HMV has been described in detail, characteristics [5, 6] of the current population using HMV have not been studied. Although HMV is still rare in children we hypothesized changes in indications and type of support over the years. As these children often survive into adulthood knowledge on these current characteristics is important for both adult and pediatric teams.This study therefore aimed to evaluate the changes in the type of support over the last four decades, and the clinical and demographics characteristics.

## Methods

We conducted a retrospective, single-center study, performed at the center of HMV at the University Medical Center Utrecht (UMCU). This center provides care for approximately 45% of Dutch children who require HMV. We included all children who used HMV between January 1979 and January 2023 and retrieved data from a prospective HMV database and medical notes including ventilation characteristics, diagnosis, reason for and age at initiation of HMV and type of ventilation. HMV was started either electively, i.e. during a planned admission to start HMV because of diagnosis of chronic respiratory failure (CRF), or unplanned, i.e. during an intensive care admission with respiratory failure with unsuccessful weaning. This is mostly caused by infection or after surgery. Follow-up data were analyzed until discontinuation of HMV, death or transfer of care to another HMV center. In case children continued care in our adult HMV center, we also analyzed adult follow-up data.

The Dutch HMV registry distinguishes 5 different categories of diseases necessitating initiation of HMV: 1. neuromuscular disorders (NMDs); 2. thoracic cage disorders; 3. lung diseases; 4. sleep related breathing disorders; and 5. other causes [3, 7]. Because we experienced that this categorization is not sufficient for children with CRF, we decided to modify these categories: 1. Upper airway diseases (UAD); 2. Congenital central hypoventilation syndrome (CCHS); 3. Central nervous system (CNS) diseases; 4. Spinal cord pathology (SCP); 5. NMDs; 6. Malignancies; 7. Lung diseases; 8. Genetic syndromes; 9. Thoracic cage disorders; and 10. Other causes. If patients fitted in multiple categories, we registered the most important reason for initiating HMV. This study does not fall under the scope of the Dutch Medical Research Law Involving Human Subjects Act. We received a waiver from the medical ethics committee at the UMCU. A scientific quality officer at the UMCU performed an independent quality check to ensure compliance with legislation and (local) regulations.

### Statistics

We used SPSS29.0.0 for data analysis. All variables were tested for normality. We calculated mean and standard deviation (SD) for normally distributed variables and median and interquartile ranges (IQR) for parametric data. We used independent samples t-test for normally distributed variables and Mann–Whitney U test for parametric data to compare groups. We used Chi-square test to compare more than 2 groups for categorical variables and Kruskal Wallis test for comparison of parametric continuous data.

## Results

### Patient characteristics

During the study period 544 children started HMV at a median age of 13.1 years (IQR 5–16). Nearly 40% of patients started HMV unplanned, because of unsuccessful weaning after surgery or an infection. One third of all children started with IPPV. Half of them had a NMD. Patient characteristics are summarized in Table 1.

**Table 1:** Patient characteristics classified by causes of chronic respiratory failure

Disease category	Number of patients, n (%)	Age *, Median (IQR)	Male gender, n (%)	Electively HMV*, n (%)
Total cohort	544 (100)	13.1 (5–16)	383 (70)	336 (62)
Congenital central hypoventilation syndrome (CCHS)	26 (5)	2.9 (0.7–7.1)	14 (54)	10 (39)
Central nervous system (CNS)	7 (1)	14.6 (10.8–17.6)	3 (43)	4 (57)
Spinal cord pathology (SCP)	39 (7)	13.5 (7.6–16.2)	22 (56)	18 (46)
Neuromuscular disorders (NMD)	305 (56)	14.7 (9.9–16.7)	243 (80)	206 (68)
Malignancy	17 (3)	13.7 (8.5–14.8)	8 (47)	8 (47)
Lung diseases	47 (9)	8.7 (0.7–14.9)	27 (57)	22 (47)
Syndrome	85 (16)	6.3 (1.2–12.1)	54 (64)	57 (67)
Thoracic cage disorder	6 (1)	14.8 (9.7–16.8)	4 (67)	4 (67)
Upper airway disease (UAD)	9 (2%)	0.9 (0.8–2.5)	5 (55)	6 (66)
Various	3 (1%)	1.6 (0.7–10.6)	3 (100)	0

*Legend*: *HMV*, home mechanical ventilation, *NIV* non-invasive ventilation, *: at start of HMV

### Age at start of HMV

Children started with HMV at an increasingly younger age. The relative number of children younger than 5 years using HMV increased significantly, from 13% in 1989–1998 to 30% in 2014–2022. This increase was even more pronounced in children using NIV: from < 4% before 2003 to 30% in 2019–2022. The median age at initiation of HMV differed significantly between disease categories. Children with UAD (0.9 years, *n* = 9) and CCHS (2.9 years, *n* = 26) were significantly younger than those in the other groups (*P* < 0.05).

### Outcome

Thirty percent (*n* = 167) died during follow-up, 66 cases during childhood. Mortality was 63% in cases who started HMV before 2000 (*n* = 63/100), compared to 23% in cases who started HMV (*n* = 104/444) from 2000 onwards. Transition to adult care took place in 253 (47%) children, while the care of 22 children was transferred to another HMV center. Totally, 131 (24%) children discontinued HMV during childhood, due to not accepting and removing the mask (*n* = 9, 7%), imbalance between benefits and burden (*n* = 35, 27%) or transient hypoventilation (*n* = 87, 66%).

### Detailed description of different disease categories

**Fig. 1:**
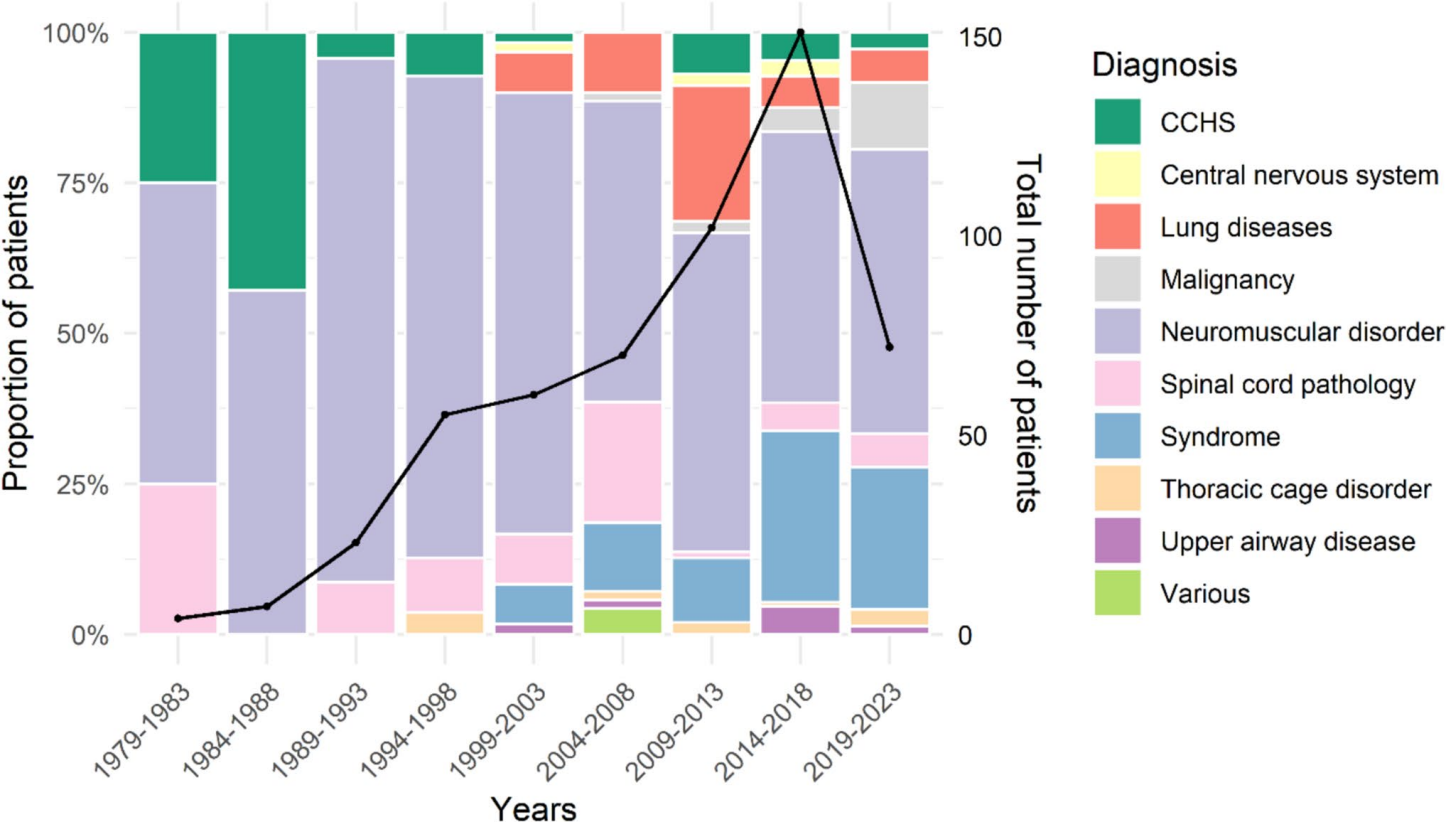
Relative distribution of diagnoses per year group among all ventilated patients (left axis). The black line and dots represent the total number of patients per year group (right axis)

NMD and SCP remained the main reason for starting HMV, but from 2000 other indications gradually increased (Fig. 1).

#### Neuromuscular disorders (NMDs)

Children with NMDs represent the largest group (56%). Table 2 summarizes patient characteristics. Eighty-three (27%) children started with IPPV and 222 (73%) children with NIV (Table 3). Seven children had a tracheostomy before start of ventilation because of difficulties in airway clearance (*n* = 2) or because it was standard care before scoliosis surgery until the early nineties (*n* = 5). Four patients with IPPV (5%) could be weaned to NIV at a median age of 16.3 years (IQR 9.0–25.7). Sixty-one children who initially used NIV switched to IPPV in adolescence or early adulthood (27%), usually due to difficulties with airway clearance and/or daytime CRF. Median age at tracheostomy was 18.0 years (IQR 15.4–20.7). Half of these patients had their tracheostomy before the introduction of mouthpiece ventilation in 2003.

**Table 2:** Overview of various neuromuscular disorders

Type of NMD	Specific diagnosis	Number of patients, n (%)	Electively started HMV n (%)	Age *, median (IQR)	IPPV *, n (%)
Congenital myopathy	Nemaline myopathy, multi mini core, RYR1 mutation	45 (15)	25 (55)	10.5 (2.2–15.0)	18 (40)
Metabolic myopathy	Mitochondrial myopathy, Pompe’s disease	18 (6)	11 (61)	9.7 (7.9–13.7)	5 (27)
Neuromuscular junction disorder	Myasthenic syndrome, myasthenia gravis	4 (1)	3 (75)	9.7 (8.5–13.4)	1 (25)
Neuropathy	HMSN, Guillain-bare syndrome, Giant cell neuropathy	15 (5)	11 (73)	14.6 (9.8–15.6)	5 (33)
Muscular dystrophy	Duchenne, limb girdle, Becker, Ulrich	164 (54)	125 (76)	16.0 (14.5–17.3)	35 (21)
Motor neuron	SMA, Brown, Vialetto van Laere, SMARD, AFM	59 (20)	31 (53)	9.5 (1.9–13.2)	19 (32)

*Legend*: *AFM* Acute Flaccid Myelitis, *HMSN* Hereditary Motor and Sensory Neuropathy, *SMA* Spinal Muscular Atrophy, *SMARD* Spinal Muscular Atrophy Respiratory Distress, *: at start of home mechanical ventilation

**Table 3:** Type of HMV support classified by causes of chronic respiratory failure

	Total		NIV			IPPV	
Disease category	n (%)	n (%)	Age*, median (IQR)	Transition to IPPV, n (%)	n (%)	Age*, median (IQR)	Wean to NIV
Total cohort	544 (100)	371 (68)	13.8 (8.5–16.1)	66 (18)	173 (32)	5.4 (0.9–15.3)	22 (13)
Congenital central hypoventilation syndrome (CCHS)	26 (5)	12 (46)	7.8 (4.3–12.6)	0	14 (54)	0.7 (0.2–2.1)	8 (57)
Central nervous system (CNS)	7 (1)	3 (43)	12.7 (10.3–15.3)	0	4 (57)	15.7 (14.6–17.2)	0
Spinal cord pathology (SCP)	39 (7)	17 (44)	10.0 (7.4–15.2)	1 (6)	22 (56)	14.0 (9.6–16.8)	2 (9)
Neuromuscular disorders (NMD)	305 (56)	222 (73)	14.9 (10.6–16.8)	61 (27)	83 (27)	14.3 (2.7–16.2)	4 (5)
Malignancy	17 (3)	12 (71)	13.8 (10.3–16.8)	1 (8)	5 (29)	9.3 (3.7–14.6)	0
Lung diseases	47 (9)	27 (57)	14.5 (12.0–16.0)	0	20 (43)	0.6 (0.4–0.8)	1 (5)
Syndrome	85 (16)	66 (78)	8.3 (2.9–13.2)	1 (2)	19 (22)	0.9 (0.4–0.9)	2 (11)
Thoracic cage disorder	6 (1)	6 (100)	14.8 (9.7–16.8)	1 (17)	0	n/a	n/a
Upper airway disease (UAD)	9 (2)	4 (45)	1.7 (0.9–2.5)	0	5 (55)	0.9 (0.6–1.1)	0
Various	3 (1)	2 (67)	6.1	0	1 (33)	0.7	0

*Legend*: *HMV* home mechanical ventilation, *n*: number, *n/a*: not applicable, *NIV* non-invasive ventilation, *IPPV* invasive positive pressure ventilation, *IQR* interquartile range, *: at start of HMV

#### Congenital central hypoventilation syndrome (CCHS)

A total of 26 children started with HMV because of CCHS due to PHOX2B mutation (*n* = 19), Rapid-onset Obesity with Hypothalamic dysfunction Hypoventilation and Autonomic Dysregulation (ROHHAD) (*n* = 3) or an elusive diagnosis (*n* = 4). None of these 26 patients could be weaned. Three patients died. Fourteen (54%) children started with IPPV (Table 3). Eight patients (57%) could be weaned to NIV, at a median age of 11.5 years (IQR 8.6–14.1).

#### Central nervous system (CNS) diseases

Seven children with cerebral palsy or CNS damage after cerebrovascular accidents or infection, started with HMV at a median age of 14.6 years (IQR 10.8–17.6). Four (57%) used IPPV (Table 3). Three children (43%), could be weaned. One patient died after 16 years of HMV.

#### Spinal cord pathology (SCP)

Thirty-nine children had SCP: spina bifida (*n* = 22, 56%) or traumatic spinal cord injury (SCI) (*n* = 17, 44%). Sixteen (73%) children with spina bifida started on NIV, one of them required tracheostomy after a few months. Two invasively ventilated children could wean to NIV. Two children with SCI (12%) started on NIV. Seven (41%) children with SCI could be weaned.

#### Malignancy

Seventeen children required HMV because of cancer. Most of these patients suffered from CRF caused by the primary tumor, such as neuroblastoma causing diaphragm paralysis and CNS tumors causing apneas and hypoventilation (65%). In the other patients, CRF was secondary to treatment, caused by muscle weakness or lung fibrosis. The median age at initiation of HMV was 13.7 years (IQR 8.5–14.8). Twelve out of 17 (71%) started with NIV (Table 3). Mortality was 18% (*n* = 3). Six (35%) children could be weaned.

#### Lung diseases

Forty-seven children required HMV because of primary lung diseases (Table 4). Six (13%) children required HMV because of bronchopulmonary dysplasia (BPD). Three (50%) of these children died.

**Table 4:** Characteristics of children who started HMV because of lung diseases

	n	IPPV, n (%)	Age in years at start of ventilation, median (IQR)
BPD	6	5 (83)	0.7 (0.4–0.9)
CF	16	0 (0)	15.9 (14.7–16.0)
Congenital cystic lung disease	2	0 (0)	7.3 (3.4–10.7)
ILD	10	2 (22)	10.27 (4.3–13.9)
Pulmonary hypoplasia	11	11 (100)	0.73 (0.4–0.8)
Other	2	2 (100)	0.3 (0.2)

*Legend: BPD* bronchopulmonary dysplasia, *CF* cystic fibrosis, *ILD* Interstitial Lung disease

Sixteen (34%) teenagers with Cystic Fibrosis (CF) received NIV as a bridge to transplant. Ten received lung transplantation, 3 died on the waiting list and 3 children discontinued NIV because of limited benefit (n = 2) or improved condition with new drugs. Since the introduction of new treatments in 2014 only two children started HMV. Two children (4%) with congenital cystic diseases required NIV. One child was transplanted successfully, the other could be weaned after 6 months. Ten children (21%) were diagnosed with interstitial lung disease (ILD), due to metabolic diseases (70%), bronchiolitis obliterans (10%), primary immunodeficiency (10%) or sickle cell disease (10%). Seventy percent of these children had a stem cell transplantation. Mortality in this group was 50%.

Eleven (23%) infants were diagnosed with pulmonary hypoplasia. One child died, the other 10 children could be weaned and tracheostomy was closed at a median age of 4.1 years (3.1–4.6).

The group with other lung diseases included two neonates requiring IPPV from birth because of Primary Ciliary Dyskinesia (PCD) and small airway disease. They could be weaned after 14 months and 6 years respectively.

#### Syndromes

Eighty-five children were diagnosed with a specific genetic or dysmorphic syndrome. Children in this group started HMV at a median age of 6.3 years (IQR 1.2–12.1). We subclassified these patients in the following categories: central hypoventilation (*n* = 3), obstructive hypoventilation (*n* = 47), mixed hypoventilation (*n* = 15) and restrictive lung function (*n* = 20). Three patients with central hypoventilation required NIV from a median age of 11.4 years (IQR 6.3–13.6). Two of these children, died. Fifteen (18%) children had mixed hypoventilation, mostly due to achondroplasia (*n* = 8). Median age at start of HMV in this group was 2.6 years (IQR 0.9–6.0). One child with achondroplasia died. Five children discontinued HMV. Forty-seven patients (55%) with obstructive hypoventilation started HMV, because of Down syndrome, or craniofacial syndromes such as Pierre Robin syndrome or Treacher Collins syndrome. Four children died. Five patients with IPPV (38%) discontinued HMV. Twenty (24%) children had hypoventilation due to restrictive lung function. This group included children with syndromes with skeletal involvement. Median age at start of HMV was 13.5 years (IQR 9.0–15.7). Five patients died. Three patients discontinued ventilation, because of transient hypoventilation (*n* = 1) or no improvement of symptoms (*n* = 2).

#### Thoracic cage disorder

This group included 6 children with scoliosis and/or kyphosis not due to a NMD. One patient discontinued HMV, as he experienced no improvement. All started with NIV (Table 3). One patient intensified to IPPV due to increased ventilatory requirements.

#### Upper airway disease (UAD)

Nine children initiated HMV because of UAD between 2000 and 2019 at a median age of 0.9 years (IQR 0.8–2.5). These children were diagnosed with tracheobronchomalacia, in some cases combined with esophageal atresia or laryngeal cleft. Four (44%) children started NIV (Table 3). Two children discontinued NIV after 1 year. Four children with IPPV, could be weaned after 2.0 years (IQR 1.2–2.9).

#### Various diagnoses

In this miscellaneous group (*n* = 3) HMV was needed because of pulmonary hypertension, juvenile dermatomyositis or diaphragm paresis after cardiac surgery. They all started in a semi-acute setting after a long period of ventilation on the ICU at a median age of 1.7 years (IQR 1.2–6.1). They all could be weaned after 7.2 years (IQR 4.7–10.3).

### Ventilation techniques over the years: non-invasive versus invasive ventilation

**Fig. 2:**
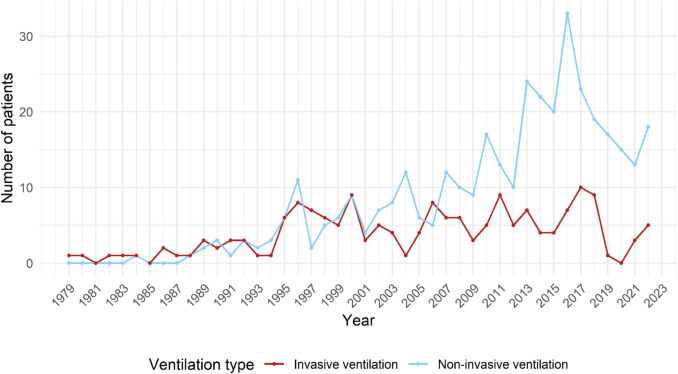
Trends in the number of children starting non-invasive and invasive ventilation over time

HMV started as negative pressure ventilation, followed by IPPV from the 1980s. NIV became available from 1990 onwards. For the last 15 years NIV was the predominant mode in children starting HMV (Fig. 2).

One-third (*n* = 173) of children started with IPPV, of which 66 (38%) were infants. Patients who started with IPPV (median age 5.4 years, IQR 0.9–15.3) were significantly younger than patients who started NIV (median age 13.8 years, IQR 8.4–16.1), (*P* < 0.001). NIV was commonly started electively (83%), in contrast to IPPV (17%). IPPV was chosen when the child was very young, had very limited ventilator free time, facial problems, or problems with airway clearance. Eleven (6%) patients had a tracheostomy prior to initiation of HMV, because of scoliosis surgery (*n* = 4), airway clearance problems (*n* = 3), or malacia (*n* = 4). Fifty (29%) tracheostomy patients could be decannulated. Eleven of these patients (22%) still needed NIV. Median age of weaning to NIV was 12.8 years (IQR 8.8–19.4).

Of the 371 patients who started with NIV, 65 (18%) intensified to IPPV because NIV was no longer effective or because of difficulties with airway clearance. These were mainly patients with NMDs (94%). Ten children who initially did not accept NIV, started successfully at a later stage. These patients were children who did not tolerate NIV, when HMV was initiated prior to surgery.

## Discussion

This large longitudinal cohort study shows, after two periods of rapid growth, a stabilization in the number of children requiring HMV. The causes of CRF necessitating HMV in children, changed over the years and are different from adult patients. A minority of children using HMV (11%) could be weaned, while 18% who started with NIV eventually had to be intensified to IPPV. Although HMV remains an intervention that is used infrequently, there is a rapidly growing group of adult patients on HMV [3]. Our study shows a different trend among children, since the number of children using HMV remained relatively stable over the last decade. In January 2023, 262 children in the Netherlands used HMV, corresponding to a prevalence of 7 per 100,000 children [8, 9]. Similar trends of stabilization have been observed in the United Kingdom and Canada [10, 11]. Dutch demographic data show a continuing decrease in the number of people younger than 20 years living in the Netherlands (15% decline between 1980 and 2022) [8], indicating that the observed growth in children using HMV in this time period was primarily caused by an extension of indications for HMV. Although the current list of indications may be inclusive, the introduction of new disease specific therapies may further change the indications for HMV in the future. An example are the genetic therapies for spinal muscular atrophy (SMA) [12] that were introduced between 2017–2023 and altered disease course. This led to changes in standards of care including HMV, particularly for the most severe phenotype (type 1). Until then, HMV was not offered to parents of children with SMA type 1. The example of CF, where the introduction of disease-specific treatments resulted in postponing respiratory failure, shows that therapies may also lead to fewer patients needing HMV. The broadening spectrum of diseases was made possible by the introduction of NIV in the 1990s. Since then, the number of children receiving IPPV has decreased [13–15]. In the last decades NIV can be more often used successfully in patients with compromised airway clearance, due to the introduction of airway clearance techniques such as airstacking and mechanical insufflation-exsufflation [16, 17]. The introduction of mouthpiece ventilation led to a further decrease in the number of patients using IPPV [18]. In very young children IPPV is used relatively common, as chronic NIV may result in midface problems [19]. Also, leakage and accepting the mask are challenges at a young age. Leakage can sometimes be solved by using a pacifier, chin strap or better-fitting masks. Increased use of NIV, even in young children, indicates that these strategies are often successful. IPPV may be safer in young children who are highly dependent on ventilation, for instance in children with CCHS. Most children (*n* = 305 (56%)) had NMDs, followed by genetic and/or dysmorphic syndromes (15%) causing airway obstruction and/or central hypoventilation. The large number of patients with NMDs is comparable to adult population and other pediatric studies [10, 11, 13]. The number of children with genetic and dysmorphic syndromes is increasing over the years as described by Tan [14]. The transition of these children to adult care requires expertise on the complex comorbidities [10]. Mortality of patients who started HMV during childhood was 30%, which is higher than in other studies [10, 20, 21]. This is explained by inclusion of patients from the 1980 s onwards, the higher percentage of NMDs, and follow-up into adulthood [10, 15, 20]. Acceptance rates of HMV were very good. In our cohort, < 2% of patients did not accept HMV initially and HMV was reintroduced successfully later. This is less than described in other studies [22]. Timing of initiation of HMV is crucial because starting prior to the onset of symptoms of CRF results in poor acceptance.

Our study has some strengths. It is the largest longitudinal cohort study of children using HMV, with a long follow-up and well characterized population. Other studies were smaller (maximum 496 children), cross-sectional or longitudinal with limited follow-up [10, 11, 20]. There were no missing data, due to standardized data documentation. The results of this study are generalizable to the Netherlands as care for patients using HMV is protocolized and uniform between all centers [7]. A limitation of the study is that data on quality of life and adverse events (mid face problems, pressure sores) are missing. These data are important to evaluate the balance between burden and benefits of HMV. HMV in children is not only technically challenging, but also ethically. The diagnosis and prognosis are not always clear at the time a decision on starting HMV is required. Increased costs and shortage of home care professionals force us to prioritize within this complex HMV care. For this reason, it is important to collect data on outcome, especially in children with more rare diseases as the available literature is limited.

## Conclusion

The number of children with HMV increased from the 1980 s but stabilized in the last decade. For the last 15 years NIV was the predominant mode in children starting HMV. The relative number of children younger than 5 years using HMV increased significantly over the years. This increase was even more pronounced in children using NIV. While the spectrum of indications has changed over the years, NMDs are still the most common reason for starting HMV in children. Adult teams must prepare to take care of a new group of patients with syndromal or genetic diseases, as many of these children survive into adulthood.

## Data Availability

The data that support the findings of this study are not openly available due to reasons of sensitivity and are available from the corresponding author upon reasonable request.

## Abbreviations

CCHS: Congenital central hypoventilation syndrome
CNS: Central nervous system
AFM: Acute flaccid myelitis
BPD: Bronchopulmonary dysplasia
CF: Cystic fibrosis
CRF: Chronic respiratory failure
HMV: Home mechanical ventilation
HMSN: Hereditary Motor and Sensory Neuropathy
ICU: Intensive care Unit
ILD: Interstitial lung disease
IPPV: Invasive positive pressure ventilation
IQR: Interquartile range
n: Number
NIV: Non-invasive ventilation
NMD: Neuromuscular disorder
PCD: Primary ciliary dyskinesia
ROHHAD: Rapid onset Obesity with Hypothalamic dysfunction, Hypoventilation and Autonomic Dysregulation
SCI: Spinal cord injury
SD: Standard deviation
SCP: Spinal cord pathology
SMA: Spinal muscular atrophy
SMARD: Spinal muscular atrophy respiratory distress
UAD: Upper airway disease
UMCU: University Medical Center Utrecht
